# From inspiration to impact: delivering value from global root research

**DOI:** 10.1093/jxb/erw215

**Published:** 2016-06-03

**Authors:** Gregory J. Rebetzke

**Affiliations:** CSIRO Agriculture

A robust understanding of root systems biology exists as one of the greatest challenges to plant and crop scientists. The complex interactions of the root with both the soil and microbiome, the dynamic and spatial complexity of root activity in both time and space, and our inability to reliably capture and predict repeatable phenotypes (Watt *et al.*, 2013) has slowed delivery and impact in root research. Yet, despite these challenges, significant efforts over the past 30 years has delivered invaluable understanding (see references herein) and, in some cases, new root traits for use in breeding improved adaptation to soil constraints (Beebe *et al.*, 2006; Ryan *et al.*, 2009). There is little question about the high quality of much of the below-ground research and understanding in the root-research community. Previous uncertainties in phenotypic complexity and robustness, the identification of appropriate targets, and clarity around complex genetic controls are now much better understood. Together, the vision of ‘roots of a new green revolution’ is being acknowledged by commercial companies who are focused on trait value in prioritizing breeding targets in selection of root traits (e.g. Reyes *et al.*, 2015).

This special issue of the *Journal of Experimental Botany* features a number of key invited review and research papers delivered at the 9^th^ Symposium of the International Society for Root Research which took place in Canberra, Australia on 6–9 October 2015. The meeting was appropriately titled ‘Roots down under: below-ground solutions to global challenges’, and represented root researchers spanning the continuum from molecular biology and modelling through to genetic understanding and commercial delivery. Additional papers relevant to the special issue and submitted separately to the *Journal of Experimental Botany* were included to complete this special issue.

The challenges of researching a dynamic system characteristic of the rhizosphere are not well understood. The thin layer of soil represented by the rhizosphere represents significant chemical, physical, and microbial interactions reflecting the interplay with the host plant and, specifically, the genotype. York *et al.* (2016*a*) review current understanding of the rhizosphere with the view of developing a conceptual framework that integrates across methodologies and disciplines to facilitate improved communication and collaboration across disparate research communities. In their second paper, York *et al.* (2016*b*) acknowledge and then investigate opportunities in the genetic improvement of nitrogen uptake and acquisition efficiency throughout the complexity of a typical maize root system. Detailed phenotyping of nitrate uptake kinetics in different root segments provided information for modelling and sensitivity analysis of the plant biomass response to changes in maximum uptake rate and transporter affinity. The potential exists to use breeding and high-throughput phenotyping to maximize uptake rates across all root architectural phenes, and to deliver greater N acquisition and increased shoot biomass.

Plant nutrients are costly to apply and can contribute to environmental pollution when oversupplied or used in soils prone to leaching. Improved nutrient uptake, giving rise to greater nutrient-use efficiency, is an objective in some crop breeding programmes. In their review, Wissuwa *et al.* (2016) highlighted the opportunity and capacity to identify and deliver phenotypes and molecular markers for key root nutrient-uptake traits into rice breeding programmes. Various transporters have been identified in the uptake of macro- and micronutrients in rice. In their review, Sasaki *et al.* (2016) describe the distinct nature of the anatomy of the rice root and the resulting unique quality of the influx and efflux transporters required in mineral transport from the root to the stele. Yet, despite this molecular and physiological understanding, there is still significant knowledge required of other mineral transporters.

The challenges afforded to careful and robust root phenotyping has obstructed efforts to link the above- and below-ground understanding of phenology. Radville *et al.* (2016) examine the importance of characterizing root growth and development with a changing climate. They highlight that roots respond differently to shoots and that the separate factors contributing to root architecture may themselves respond differently to environmental changes that are independent of those influencing the growth of the shoot.

Acid soils are commonplace throughout cropping regions of the globe. Deficiencies and toxicities associated with acid soils reduce shoot growth but the response is first recognized by the root, particularly in the inhibition of primary roots while lateral root growth is promoted. The complex response by roots to acid soils is reviewed in Sun *et al.* (2016) and provides insight into how plant hormones (primarily auxin and ethylene) are key in co-ordinating the root response to phosphorous deficiency and aluminium toxicity which are common to acid soils. Understanding through genetic control may provide opportunities for targeted breeding to improve root performance in acid soils.

Identifying target root phenotypes for selection in breeding programmes can be challenging, particularly in variable rain-fed, environments. Lilley and Kirkegaard (2016) investigated the influence of different root architectures and sowing date on wheat yields using simulation models for soil types and contrasting agronomic systems across the Australian wheat belt. Using historic rainfall data, the authors concluded that the benefit of a more extensive root system was greater in deeper soils where there was the capacity to capture and use deep soil-water. Extensive root systems were of little benefit in shallow soils where greater water use and yield was associated with wheat crops sown early. The use of modelling demonstrates the capacity to identify potentially useful ideotypes prior to the expense of detailed population development and phenotyping.

Linking complex below-ground phenotypes with information on genetic diversity was a key theme in Chen *et al.* (2016) for understanding the opportunities of breeding improved narrow-leafed lupin varieties for rain-fed systems. A diverse range of root traits and root trait combinations were identified and then genotyped with molecular markers in this diverse group of lupin accessions. Marker-trait associations revealed opportunities for marker-assisted selection in the development of lupin genotypes with improved performance across a broad range of potential environments.

Salinity is of growing concern globally and the paper by Robin *et al.* (2016) highlights that the response to salt occurs in both the roots and the shoots. In carefully-designed experiments, wheat roots exposed to increasing salt concentrations were reduced in both root hair length and root hair density with root hairs contributing up to 90% of the total root surface area. Interestingly, changes in individual root traits varied among wheat genotypes suggesting that there were opportunities for aggregating traits via breeding new varieties with improved salinity tolerance.

Hypoxia arising from prolonged periods of plant water-logging can slow root and shoot growth to reduce biomass and grain yield in many crops. Wang *et al.* (2016) highlight the key role of Ca^2+^ during water-logging in a series of detailed physiological studies investigating several knock-out Arabidopsis mutants subjected to several days of controlled water-logging. The work highlighted the impact of water-logging and hypoxia on reductions in chlorophyll fluorescence and subsequent shoot biomass, and that calcium transporters played a key role in regulating the hypoxic responses.

The metabolic pathways implicated in the tolerance to salinity stress were explored in studies by Shelden *et al.* (2016). In controlled studies comparing salt-tolerant and salt-sensitive barley varieties, metabolic pathways linked to the increased synthesis and accumulation of amino acids contributed to the maintenance of cell division and root elongation. Further, this control appeared to be co-ordinated and region-specific along the salt-challenged root.

Root rhizosheaths can significantly extend root surface area in order to increase phosphorous uptake in low or phosphorous-deficient soils. Genetic diversity for the rhizosheath trait in wheat has highlighted the opportunities in breeding for this trait in cereals. James *et al.* (2016) investigated near-isogenic lines and populations varying for root rhizosheath size in order to establish an increase in shoot biomass with large rhizosheaths on acid and low soil phosphorous-containing soils. Using structure populations, large effect QTL and genes of additive genetic effect were identified suggesting marker-based and phenotypic selection should deliver breeding lines with large rhizosheath size in early generations of a breeding programme targeting improved phosphorous uptake. The broader theme of root hair formation and its importance in phosphorous uptake was extended to rice in Nestler *et al.* (2016). Root hair phenotypes in the field did not translate well to assessment in hydroponic systems indicating a limitation to testing in nutrient solutions. This is understood to reflect differences in root hair formation across the different lateral root types.

Moving away from crops and into natural systems, the complex and diverse chemistries of compounds produced in the root hairs of successful invasive weeds is elegantly explored in Zhu *et al.* (2016) for the invasive pest *Echium plantagineum*. Bioactive shikonins implicated in plant defence and invasion success were produced soon after germination in the root–hypocotyl junction and in root exudates. These shikonins were subsequently extracted from living roots and in the rhizosphere and bulk soil surrounding the roots demonstrating the rhizodeposition of this compound and its implication in invasion and colonization.

This unique collection of research and review papers demonstrates the capacity and understanding of root science in natural plant communities through to intensive cropping systems.
